# Extended and Structurally Supported Insights into Extracellular Hormone Binding, Signal Transduction and Organization of the Thyrotropin Receptor

**DOI:** 10.1371/journal.pone.0052920

**Published:** 2012-12-27

**Authors:** Gerd Krause, Annika Kreuchwig, Gunnar Kleinau

**Affiliations:** 1 Leibniz-Institut für Molekulare Pharmakologie, Berlin, Germany; 2 Institute of Experimental Pediatric Endocrinology, Charité Universitätsmedizin Berlin, Berlin, Germany; University of São Paulo, Brazil

## Abstract

The hormone thyrotropin (TSH) and its receptor (TSHR) are crucial for the growth and function of the thyroid gland. The TSHR is evolutionary linked with the receptors of follitropin (FSHR) and lutropin/choriogonadotropin (LHR) and their sequences and structures are similar. The extracellular region of TSHR contains more than 350 amino acids and binds hormone and antibodies. Several important questions related to functions and mechanisms of TSHR are still not comprehensively understood. One major reason for these open questions is the lack of any structural information about the extracellular segment of TSHR that connects the N-terminal leucine-rich repeat domain (LRRD) with the transmembrane helix (TMH) 1, the hinge region. It has been shown experimentally that this segment is important for fine tuning of signaling and ligand interactions. A new crystal structure containing most of the extracellular hFSHR region in complex with hFSH has recently been published. Now, we have applied these new structural insights to the homologous TSHR and have generated a structural model of the TSHR LRRD/hinge-region/TSH complex. This structural model is combined and evaluated with experimental data including hormone binding (bTSH, hTSH, thyrostimulin), super-agonistic effects, antibody interactions and signaling regulation. These studies and consideration of significant and non-significant amino acids have led to a new description of mechanisms at the TSHR, including ligand-induced displacements of specific hinge region fragments. This event triggers conformational changes at a convergent center of the LRRD and the hinge region, activating an “intramolecular agonistic unit” close to the transmembrane domain.

## Introduction

### Previous knowledge of the extracellular TSHR and GPHR regions

#### The general topology of GPHR structures

The general structural topology of the homologous glycoprotein-hormone receptors (GPHRs) is identical with that of other G-protein-coupled receptors (GPCRs) and is characterized by an extracellular N-terminal region, seven transmembrane helices (TMHs) connected by three intracellular loops (ICLs) and three extracellular loops (ECLs) and with an intracellular tail. The TMHs and loops constitute the serpentine domain (SD), which spans the membrane from the extra- to the intracellular site.

A common special structural feature of all GPHRs, in contrast to other family A GPCRs (rhodopsin-like), is that they contain a large N-terminal extracellular region, comprising more than 320 amino acids (reviewed in [1]). This receptor region interacts with the hormone(s) and is the initial mediator of signal transformation (reviewed in [2]). This N-terminal extracellular region can be subdivided into: i. the N-terminal tail with the signal peptide, ii. *cysteine-box 1* (C-b1) containing four interacting cysteines, which are part of iii. the leucine-rich repeat domain (LRRD) that is constrained C-terminally by disulfide bonds between two cysteines of *cysteine-box 2* and two cysteines of *cysteine-box 3* that is part of iv. the hinge region, which connects the LRRD and the transmembrane spanning domain (reviewed in [2]).

#### The endogenous hormone-ligand binds to the leucine-rich repeat domain

The major binding region for the hormones TSH, LH, CG and FSH has been identified by experimental studies on the GPHR LRRDs [3]–[5]. The receptor/hormone interactions and molecular differences between hormone/receptor subtypes have already been intensively studied [6]–[8]. Each full repeat of the LRRD comprises 20–30 amino acids. In the case of the GPHRs, each repeat contains a conserved eleven residue segment with the consensus sequence LxxLxLxx(N/C)xL (x – L, V, I, or F), that constitutes a β-strand at the hormone binding site. These β-strands are arranged as a β-sheet [9]. The hNogo-66 receptor ectodomain (PDB entry code: 1OZN [10]) has been used previously as an advanced structural GPHR LRRD template that predicted the GPHR LRRDs as shaped like a “scythe-blade” [11], rather than like a “horse-shoe”, as had been assumed [12]. This GPHR LRRD model based on the Nogo-66 receptor also indicated the N-terminal cysteine-box 1 of GPHRs as an integral part of the LRRD structure, representing an LRRD flanking ‘N-cap’ motif [13], [14]. Furthermore, this TSHR LRRD model predicted that the folds are not stabilized by helices at the concave site - as it is known for other types of LRRDs [15] - but instead by interaction between aromatic side chains in the hydrophobic interior core designated as ‘Phe-spine’. Interestingly, the sequence (amino acid positions 261–281) following the previously assumed 9 full repeats [8], [16] is composed of a typical sequence pattern of leucine-rich repeats, indicated a full tenth repeat and an eleventh β-strand on the convex surface [11]. Site-directed mutagenesis of specific residues in this region and modeling approaches supported the hypothesis of an extended LRRD through Cb-2 that interacts with Cb-3 through disulfide bridges [11], [17]. In summary, these extracellular components were predicted to be arranged in close spatial proximity, in accordance with functional findings [18]–[20].

The first crystal structure of the FSHR LRRD (published in 2005, PDB entry code: 1XWD) confirmed the predicted “scythe-blade” shape and the absence of helical secondary structural elements [21]. The N-terminal site of the LRRD was characterized by an anti-parallel β-strand and participation of Cb-1 amino acids. The C-terminus of the solved LRRD structure terminates with the tenth β-strand of an uncomplete repeat-loop around amino acid position 255 (TSHR numbering with signal peptide) (reviewed in [2]). Moreover, the structural features of 9 LRRD repeats were subsequently confirmed by crystal structure complexes between the TSHR LRRD and an activating [22] (PDB entry code: 3G04) or inactivating [23] autoantibody (PDB entry code: 2XWT), respectively.

The general spatial orientation of the hormone bound at the LRRD was also described by the complexed FSHR LRRD/FSH X-ray structure. FSH interfaces with particular β-strands from 2–9 at the concave inner surface of the LRRD [24], [25]. Thyrotropin, a ligand for TSHR, probably binds in a similar mode to that of FSH to the concave site of the LRRD. A complementary pattern of amino acid side-chain properties is responsible for specific hormone recognition [7].

#### The hinge region as a second hormone binding site also regulates signaling properties

The hinge region is located between the LRRD and the transmembrane SD. Most of the differences between the amino acids in the three homologous GPHR subtypes TSHR, FSHR, and LHR can be found in this region [1]. The cysteines of C-b2 (TSHR: Cys283, Cys284, Cys301) at the border between LRRD and the hinge region are linked by disulfide bonds to the cysteines of C-b3 (TSHR: Cys390, Cys398, Cys408) close to TMH1. Indirect evidence has permitted the global assignment of disulfide bridges between these six cysteines (reviewed in [2]).

On the basis of site-directed mutagenesis at particular amino acids and insights from deletions leading to receptor activation, it has been concluded that the extracellular hinge regions of GPHRs are necessary for stabilization of a signaling-competent basal receptor conformation [19], [20], [26]–[34]. Constitutively active GPHR variants at the extracellular site were found at pathogenic TSHR variants or can be designed via mutations (see the freely accessible web-resource and data collection for GPHRs [35]–[37]). It has therefore been proposed that the hinge region plays an important role in the regulation of receptor activity [17], [19], [26], [33], [38], including initial signal transformation via an *intramolecular signal transmitter*. Despite specific differences between the homologous GPHRs, it is commonly accepted that an *intramolecular agonistic unit* becomes activated by different triggers [19], [20], [32], [33], [39], [40]. However, the detailed molecular mechanism of receptor activation and the regulation of the state of activity at the extracellular site were not fully understood.

It has been postulated that intramolecular interactions between the extracellular region and the SD are essential for switching from basal to activated GPHR conformations (mutual cooperative effects). Deletions of the extracellular portion can lead to partial activation of the TSHR and FSHR and encouraged the suggestion that there is an extracellular “*intramolecular tethered inverse agonist*” that switches to an internal agonist during receptor activation [19], [26], [27], [40]. A key role for this intramolecular mechanism has been proposed for TSHR amino acid Ser281 in Cb-2 (corresponds to hLHR Ser277, hFSHR Ser273). This was identified by the occurrence of pathogenic activating mutations and has been studied in detail by site-directed mutagenesis [18], [41]–[43]. In a specific TSHR Cb-3 fragment, which structurally links Cb-2 to the transmembrane region with disulfide bridges, activating single-side chain substitutions have also been found [11], [44], as well as pathogenic activating deletions [45]. Moreover, *in vitro* studies have suggested that mutations at the serpentine domain affect the conformation of a closely associated hinge region which modifies ligand binding to the extracellular region [46], [47].

Several amino acids that affect hormone binding and signaling have also been detected by mutagenesis in this region between TSHR positions 288–410, such as the polar and negatively charged Glu297, Glu303, Asp382 or Asp386 [17], [48]–[53]. Noteworthy, is a sulfated tyrosine 385, which was found to have a prominent role in hormone binding to TSHR [49], [50], but also in other GPHR subtypes [39], [54].

Generally, it has been widely accepted that the hinge region is a receptor determinant that implements fine tuning of signaling with respect to basal signaling activity, constitutive activation and hormone induced signaling. There have been several reports of cooperative intramolecular interactions between receptor components [19], [30], [33], [47], [55]–[57]. Nevertheless, there have only been partial or indirect answers to two important questions: Which amino acids constitute the intramolecular interfaces between the LRRD-hinge region - serpentine domain and how are these structural parts arranged relative to each other? How does the hormone interact with the hinge region?

### Implications of the new crystal structure of the extracellular FSHR region in complex with FSH

**Figure 1:**
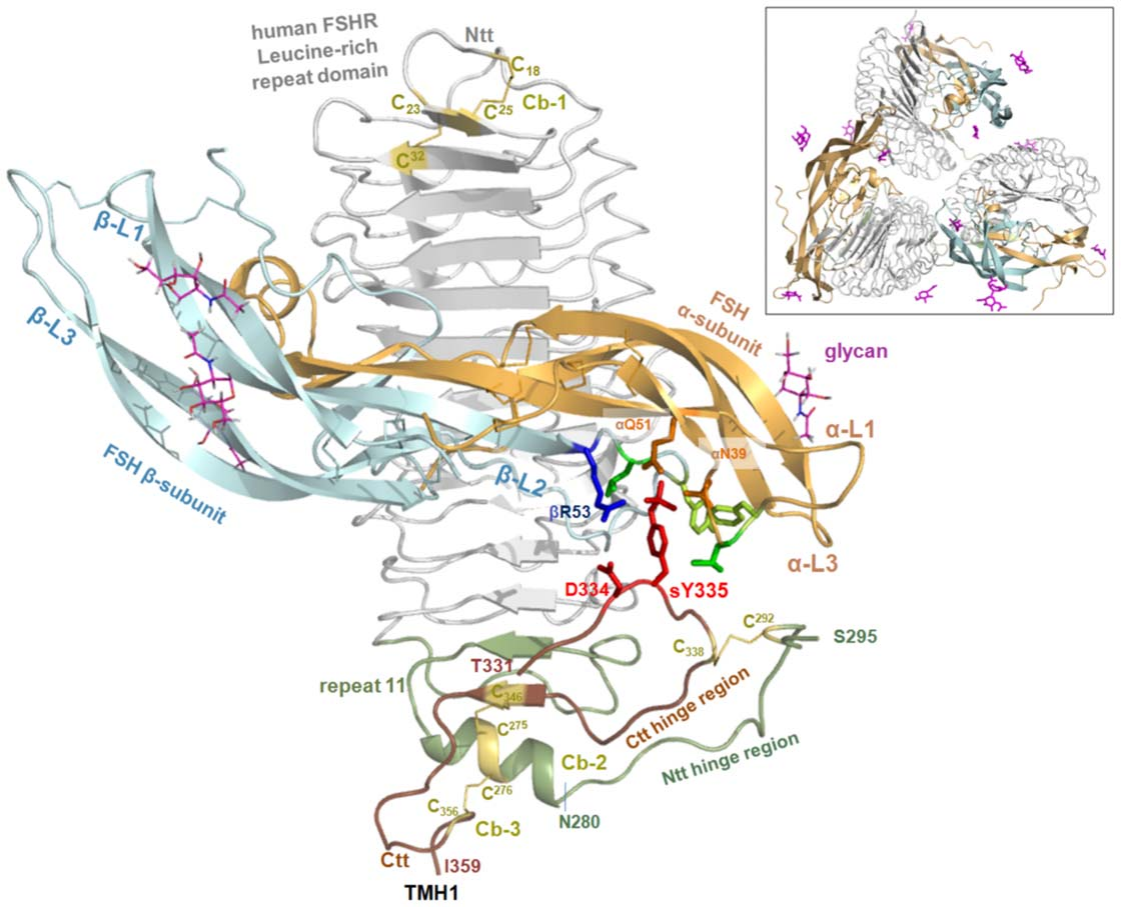
The FSHR LRRD and hinge region with bound FSH. The crystal structure of the FSHR LRRD/hinge region fragment/FSH complex is trimeric [54] (insert, boxed window). A monomeric LRRD/FSH/hinge region complex was extracted from the trimeric arrangement to visualize specific important structural and functional features. The LRRD was previously assumed to end at LRR9 with an additional tenth β-strand [21], [23]. The new structure shows an additional eleventh repeat and extends the LRRD through amino acid Asn280 (green backbone). This is followed by a short helical secondary structure, comprising the first two cysteines (Cys275, Cys276, yellow lines) of cysteine box 2 (Cb-2, N-terminal hinge region). These form disulfide bridges with cysteines (Cys346, Cys356, yellow) of cysteine-box 3 (Cb-3, C-terminal hinge region, chocolate brown), in which a short β-strand is additionally assembled parallel to the eleventh strand of the concave LRRD β-sheet. The N-terminal section of the hinge region after the LRRD is crystallized till residue Ser295. The inner cysteine bridge (Cys292-Cys338) between the two remaining extracellular cysteines connects this section with the C-terminal hinge region. It was already known from mutagenesis studies that the sulfated Tyr335 (at the C-terminal hinge region that is crystallized from position Thr331-Ile359) is mandatory for hormone binding and signaling and interacts tightly with amino acids of the hormone subunits, between the β-subunit loop 2 and the α-loop 1. The side chains of the hormone constitute a binding pocket for the sulfated tyrosine; light green sticks represent aromatic residues, green sticks hydrophobic residues and orange sticks hydrophilic residues. The adjacent negatively charged Asp334 interacts directly with the positively charged Arg53 of the hormone β-subunit. The C-terminus of the hinge region at Cys346 forms a β-strand parallel to the last repeat strand of the LRRD and ends with residues that are conserved among the GPHRs and that are known from studies on TSHR to be relevant to signaling [11], [44].

Some answers to these questions have now been provided by the recently crystallized LRRD/fragmental hinge region/FSH complex [54] (figure 1). The milestone character of this new structure is due to the addition of hinge region fragments, which were not included in the previous structure of FSHR LRR/FSH complex [21].

It is noteworthy that several aspects of hormone binding and orientation observable in the new complex were predicted 17 years earlier on the basis of a combination of functional and homologous structural information [58]. Furthermore, this FSHR crystal structure confirmed the hypothesis that the GPHR hinge region is not a self-folding domain [17]. The specific structural properties are determined by intra- and intermolecular interactions.

The new FSHR LRRD/hinge region/FSH crystal structure [54] contains trimeric receptor/ligand complexes (figure 1). The relevance of such a trimer for the natural GPHR formation should be examined in future studies, because it is well known that GPHRs form higher order complexes [47] and the extracellular region participates in oligomerization [59]. However, the previous FSHR/FSH crystal structure - with a shorter LRRD containing only 9 full repeats and without the new fragments of the hinge region [21] - has a dimeric complex which does not overlap with the recently published structure with interactions between the monomers. Experimental studies to confirm the previously observed crystal structure dimer-contacts failed to support the significance of these amino acid contacts [60]. However, this current study will mainly focus on structural information on the monomeric TSHR/ligand complex.

Beside the predicted [11] and detected [23] structural participation of TSHR Cb-1 in the N-terminal fold of the LRRD (figure 1), this new structure revealed that the LRRD continues through specific amino acids of Cb-2, with a C-terminal end at amino acid position Asn280 (figure 1). Thus, the eleven LRRs predicted by us [17] and others [12] are indeed present in this new LRRD structure. It is to be noted that previously crystallized ligand/receptor complexes contained only nine completed repeats [21]–[23], implying that the complex was already stable for protein expression and crystallization. The termination of the LRRD with a cysteine-box is consistent with a general type of LRRD architecture in which a cysteine-box represents a fold-stabilizing ‘C-cap’ motif [15]. In the particular case of the GPHRs, these cysteines are linked to cysteines of Cb-3 at the C-terminus of the hinge region. In conclusion, the previous prediction of tight spatial proximity between Cb-2 and Cb-3 [11] is also confirmed as well as the suggested cysteine-bridges [27], [28], [39]. However, the helical structure for positions 272–280 contains the two consecutive cysteines (Cys275; Cys276) that are bridged to the last two extracellular cysteines of Cb-3. They are located on one side (Cys346) at a short β-strand element parallel to the concave LRRD β-sheet 11 and on the other side (Cys356) are adjacent to transmembrane helix 1 (figure 1).The cysteine-linked helix is an integral part of the LRRD fold and this short β-strand is an extension (twelfth β-strand) of the LRRD β-sheet. Neither of them has been predicted in any previous or recent structural GPHR models.

**Figure 2:**
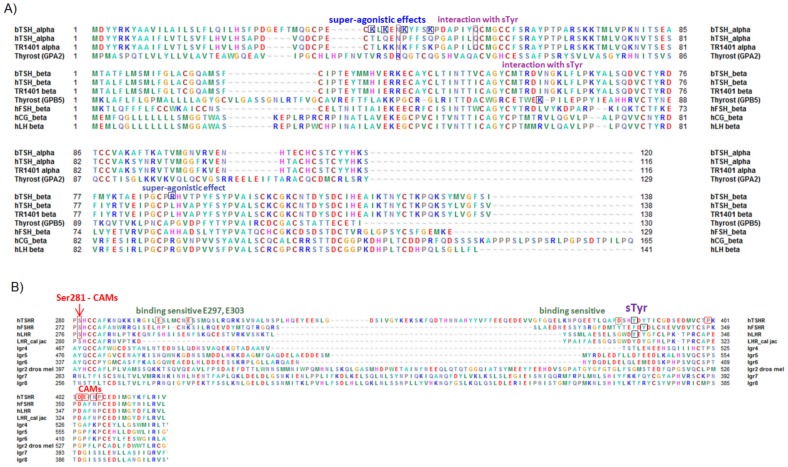
Amino acid sequences of hormone subtypes and specific receptor fragments. **A**) Alignment of hormone subtype amino acid sequences with signal peptides (numbering with signal peptides throughout the manuscript). The α-subunits of hTSH, bTSH, hTSH analogue (TR1401, Trophogen Inc., Rockville, MD) and thyrostimulin are shown, together with the β-subunit sequences of hFSH, hLH and hCG. Residues are colored according to their biophysical properties: blue - positively charged, red - negatively charged, green - hydrophobic, orange - hydrophilic, magenta – histidine, brown – cysteine, black – proline, light green - aromatic residues). **B**) Comparison of the amino acid sequences of different GPHR subtypes and LGRs. Residues are presented from the cysteine-box 2 through the transmembrane helix 1. Specificities are highlighted, including the positions of CAMs and amino acids relevant to binding.

Moreover, a specific feature of the new crystal structure is the role of a sulfated tyrosine at the C-terminus of the hinge region, as exhibited in all GPHRs (FSHR Tyr335, LHR Tyr331, TSHR Tyr385) (figure 1). Several functional studies had already confirmed the general importance of this residue for hormone binding [39], [49]. Interestingly, the homologous GPHRs in complex with their corresponding hormones (figure 2A) were suggested to have structural differences at this hormone binding-sensitive hinge region since a sulfated and binding-relevant tyrosine is common, but this is not localized at the exact position of TSHR/LHR corresponding to FSHR (figure 2B). Details of the interaction between a sulfated tyrosine and hormone are now visible in the ECD/FSH crystal structure [54] and confirm that the hinge region contains a second hormone binding site and is obligatory for conducting a signal from the extracellular site to other receptor parts [17]. The negatively charged Asp334 adjacent to Tyr335 contributes to the charge-charge hormone/receptor interactions and supports the exact adjustment of this receptor part to the hormone.

In summary, the new FSHR crystal structure of an extracellular complex between the FSHR in interaction with hFSH [54] is a milestone in GPHR research. This FSH/FSHR complex visualizes an activated state of the extracellular region. This new information supports a general subdivision of two separated binding sites at the extracellular region as follows: I. at the LRRD, II. at the hinge region around Tyr335. Binding of the hormone modifies the hinge region at specific sites, commonly at a sulfated tyrosine (TSHR Tyr385) as also shown in studies with chimeric GPHRs [39], [55]. This hormone - hinge region interplay is conveyed by a currently unknown information-pathway to the serpentine domain as it is not quite clear how the still unknown inactive conformation of the extracellular region is modified by the hormone to switch into the active conformation. On the basis of the FSHR/FSH crystal structure it has been suggested that the loop harbouring the sulfated tyrosine may be lifted [54].

What can we learn from this new FSHR crystal structure for the homologous TSHR? Due to the high sequence similarity between GPHR and their respective hormones, this new FSHR structure offers a solid basis for models of the TSHR LRRD/hinge region/ligand complex. This includes the general mode of endogenous ligand binding and detailed interactions despite known differences in GPHR-subtype specific interactions [6], [7], [48].

## Results and Discussion

On the basis of the recently published structure of the FSHR extracellular region [54] we have designed and extended our previous TSHR LRRD model with the hinge region fragments in complex with TSH, thyrostimulin and antibodies. This model answers important questions about structural and functional relations at the TSHR: How do the endogenous TSHR ligands interact with the receptor? How does the receptor become activated?

### The structural assembly of the TSHR LRRD and hinge region

**Figure 3:**
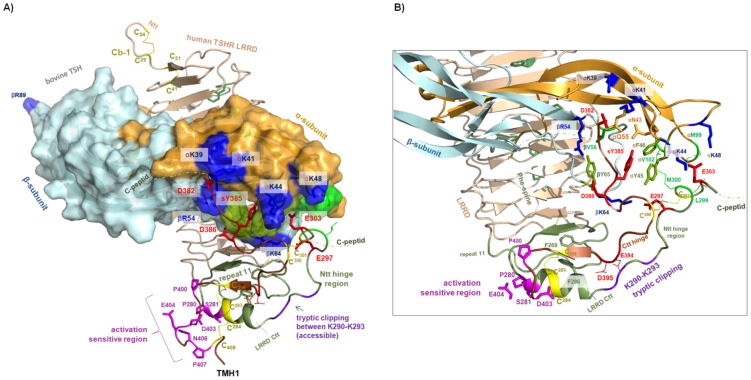
The TSHR LRRD and hinge region in complex with TSH. **A-B**) Homology model of the extracellular TSHR region complexed with bTSH. The new LRRD (pale beige backbone ribbon) repeat LRR 11 (green) contains a helical secondary element with two consecutive cysteines (yellow) of cysteine-box 2. The hormone is visualized as a surface (α-subunit in orange, β-subunit in greenblue). The sulfated tyrosine 385 (TSHR number, red stick) [49] location is comparable to that in the FSHR/FSH complex interacting with the hormone in a pocket constituted by hydrophilic (orange sticks), aromatic (light green sticks) and hydrophobic residues (green sticks). In contrast to FSH and human TSH, bovine TSH contains additional positively charged residues at the α-subunit (blue surface regions in A) and blue sticks at B)) which are known to increase hormone binding properties compared to hormone variants without those positive charges at the corresponding positions [70]. Our TSHR model suggests that these four side chains (at the α-subunit interact with negatively charged asparagines and glutamates (red sticks) in the TSHR hinge region (Asp382, Glu303) that have been shown experimentally to interplay with positively charged hormone side chains of bTSH [51], [52]. These interactions are responsible for the super-agonistic effects of bTSH compared to hTSH and are perfectly matched in this TSHR/TSH model based on the FSHR/FSH complex. Met300 and Leu299 (hydrophobic, green surface and sticks) and the negatively charged Glu297 (red stick in B) are already known to be sensitive to TSHR binding; these also have complementary partners on the hormone (details in B). Moreover, Cb-2 and C-b3 interact tightly. Amino acids at the N- and C- terminal sites (magenta) close to TMH1 (the serpentine domain) have been shown by mutation studies to activate TSHR constitutively. In Cb-2 (A), Pro280 and Ser281 at the end of helical repeat 11 region are in direct spatial proximity to amino acids Pro400–Pro407 of Cb-3. One difference between the β-subunits of bTSH and hTSH is not directly explained by the current TSHR/TSH model. The positively charged βArg89 of bTSH is absent in hTSH and substitution of this residue into hTSH leads to greatly enhanced binding of hTSH to TSHR [70], [71]. The TSHR monomer model reveals that this residue is located at the β-L3 loop and is not involved in any intra- or intermolecular interaction to the targeted receptor.

The TSHR Cb-1 constitutes the N-terminus of the LRRD as an “N-cap” motif (figure 3A). The LRRD fold is stabilized by hydrophobic and aromatic interactions localized in the interior space between the repeats. This “Phe spine” pattern also holds true for the new extension of the LRRD structure. According to the new TSHR LRRD model, the aromatic Phe269 at LRR10 interacts with Phe286 localized at the short helix as an integral part of repeat 11 (figure 3B). In support of this, mutations to non-aromatic residues at position 286 impaired the cell-surface expression of LHR [61] and TSHR signaling capacity [53] indicating that Phe286 is of structural significance.

The new TSHR model reflects the fact that the LRRD continues through specific amino acids of Cb-2 (TSHR amino acids between Cys283-Cys301) (figure 3A,B). It is important that only the eleventh repeat contains a helical secondary structure element. Initially, it had been assumed that this was the case for each repeat at the convex side of GPHR LRRDs on the basis of crystal structures of other homologous LRRDs such as the ribonuclease A inhibitor (PDB entry code 1BNH, as reviewed in [2]). This helix contains the first two cysteines of Cb-2 (Cys283 and Cys284) that are connected by disulfide bridges to Cb-3 (C-terminal hinge region) (figure 3B). Thus, bearing in mind the covalent linkage between the LRRD-Cb-2 and Cb-3 and the close proximity of the latter to the serpentine domain, this helix might be a central element and pivot of any hinge-like movement upon hormone binding or during activation. This is in line with the fact that residues Pro280 and probably also Ser281 are involved in N-terminal stabilization of this helix and are in direct spatial proximity to amino acids Pro400–Pro407 of Cb-3 (figure 3B). Several CAMs have been found, particularly at five positions in this region [11], [44] as well as mutations at Pro280 and Ser281 [18]. These wild type amino acids are highly conserved in GPHRs and have already been suggested as determinants of an *intramolecular agonistic unit* [11], [17]; it has now been confirmed that they are in close vicinity. Because Cb-3 is directly attached to TMH1, this *agonistic unit* is then localized close to the extracellular loops [11]. Interactions between these extracellular regions and the SD appear to interact cooperatively indicating tight structural interplay [19], [30], [46], [47], [55], [57]. An aromatic environment has been proposed for Ser281 and Tyr279 based on the fact that mutations of Ser281 to aromatic side-chains cause only slight receptor activation [18].

The middle of the TSHR hinge region between positions 304–381 that also includes a cleavable peptide (C-peptide, positions 317–369) [63]–[68]) is not provided in the crystal structure of the FSHR and therefore, it is missing in the TSHR model.

### Ligand binding at the extracellular TSHR region

The hormone interacts at two main receptor sites: I. at the LRRD, II. at the hinge region (figures 1 and 3A). Hormone binding at the LRRD as site I has been characterized in detail by several experimental studies [6], [7], [59]. The sulfated tyrosine 385 at hormone binding site II in the C-terminal hinge region is a key player [49], [50] as well as the negatively charged Asp386. The latter probably imitates the interaction of Asp334 in FSHR since Asp386 and not His384 (corresponding position to FSHR Asp334) has been found to be sensitive to hormone binding [53]. Therefore, in our TSH model the backbone of the sulfated tyrosine vicinity is slightly different to the FSHR structure which is in accordance to experimental findings [62]. Just as in the FSHR/FSH complex, the sulfated Tyr385 interacts with TSH in a pocket between the α- and β-subunits, which has a nearly identical composition of hydrophilic, aromatic and hydrophobic residues contributed from TSH loops α-L1 and β-L2 (figures 1 and 3).

**Figure 4:**
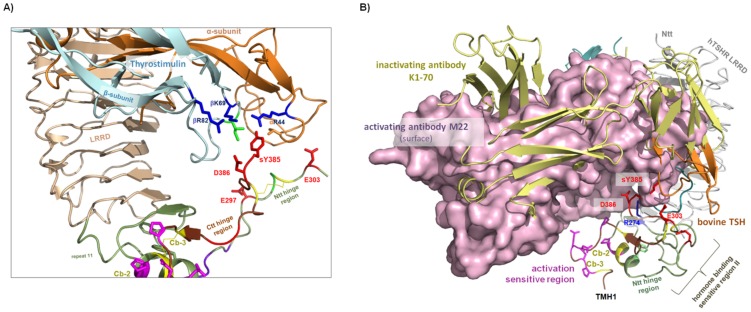
The extracellular TSHR region in complex with thyrostimulin and antibodies. **A**) The second endogenous hormone-ligand for TSHR is thyrostimulin [63]–[66] (sequence at Figure 2). Structural details of a potential heterodimeric thyrostimulin have already been described [2], [80], [81]. On the assumption that the general orientation of heterodimeric thyrostimulin at the LRRD is similar to that for bTSH (Figure 3), thyrostimulin should also interact with the sulfated tyrosine in the C-terminal hinge region. In contrast to bTSH, two positively charged residues participate in the formation of the TSHR tyrosine 385 binding site (Figure 3B), which might influence the binding properties for the sulfated negative charged sulfated tyrosine. **B**) TSHR LRRD crystal structures are available with an interacting activating [23] or inactivating [22] antibody (AB), respectively. The activating M22 (light violet surface) and the inactivating (green backbone) K1–70 bAB are bound at the concave site of the LRRD like the hormone TSH (orange and blue backbone). Relative to K1–70, the activating AB is displaced at the LRRD towards the membrane spanning part. In this study, a new extended LRRD based on the FSHR LRRD crystal structure with an additional repeat (green backbone) was modeled and structurally aligned with the antibody/LRRD structures. It is observed that M22 could interact with more residues than previously assumed, e.g. at LRR10 and 11, including Arg274 (blue stick). The activating and inactivating antibodies contact LRRD in the same general region as the hormone, but the second binding site at the hinge region seems to be hormone specific. The orientations of the hinge fragments participating in hormone binding have been identified in an active/bound LRRD/hormone complex and might therefore be differently located in TSHR/antibody-complexes. The hormone-sensitive sulfated Tyr385 should probably contact the surface of M22, but is not tightly embedded in a specific pocket. Activating antibody/receptor contacts should be relevant to the displacement of specific hinge region fragments. The new TSHR model with hinge fragments complexed with M22 predicts very tight spatial proximity between this antibody and the signaling sensitive part (activation) of cysteine-box 3 (magenta sticks). This direct interface is not observable for TSH or for K1–70.

Interestingly, a potential further endogenous ligand for TSHR is thyrostimulin [63]–[66], the ancestral hormone for glycoproteins, which is 4-fold more potent in stimulating cAMP than rhTSH [67] (sequence in figure 2A). On the assumption that the general orientation of the heterodimeric thyrostimulin at the LRRD is as suggested for bTSH we generated a model with bound thyrostimulin (figure 4A), which should also interact with tyrosine 385 in the C-terminal hinge region. However, in contrast to the sulfated tyrosine binding site of TSH the α-subunit GPA2 of thyrostimulin contains a positively charged residue (Arg44) instead of two uncharged hydrophilic residues from the hormone α-subunit (Gln and Asn from α-L1 loop).Moreover, a second positively charged residue, Lys69 from the β-L2 loop (shorter than in TSH), participates in this binding mechanism (figures 3B and 4A) and might influence binding properties.

Furthermore, for the TSHR crystal structures of the LRRD are available with either an activating [23] or an inactivating [22] autoantibody (AB). Both the activating antibody M22 and the inactivating antibody K1–70 are bound at the concave site of the LRRD like the hormone. The binding site of the activating AB M22 is displaced to the C-terminal site of the LRRD compared to the inactivating AB K1–70 (figure 4B). If we incorporate these ABs in our current TSHR LRRD/hinge region model, it is observed that the bulky body of M22 is in very tight spatial proximity to residues in Cb-3 which is sensitive for activation and also M22 might directly interact with more residues at the extended LRRD than previously assumed (figure 4B). The inactivating antibody K1–70 does not establish any contact to amino acids in the hinge region in our model.

It has to be noted here that the orientation of the hinge region fragments that participate in hormone binding was identified on the basis of an active/bound FSHR LRRD/hormone complex and might be in different locations in TSHR/antibody-complexes. However, neither the sulfated Tyr385 nor any negatively charged receptor amino acid interacts with the antibody as suggested for the hormone (figure 4B). Tyr385 should probably contact the surface of M22, but is not embedded tightly in a specific pocket. The lack of significance of Tyr385 for binding an activating antibody is in accordance with experimental findings [49]. In conclusion, these activating and inactivating antibodies contact the LRRD in an area which largely overlaps with hormone binding, but the second binding site at the hinge region seems to be specific to hormone binding. Therefore, the molecular activation mechanisms might be different in detail for M22 and TSH. The bulkier antibody should achieve activating effects by steric displacement of the hinge region fragment and directly by contact and modification of signaling sensitive hinge region fragments.

Moreover, further TSHR binding sensitive residues have been previously identified by experiments on the N-terminal hinge region including Leu299 and Met300 or the negatively charged Glu297 [52], [53], [68]. In the current TSHR/TSH model they interact with complementary (hydrophobic or positively charged) amino acids in the hormone (details in figure 3B).

Furthermore, additional signaling or binding relevant amino acids at the C-terminus of the hinge region have been suggested based on mutagenesis studies [52], [53], [68] such as Glu394 and Asp395. We speculate here in accordance to a recent experimental report [69] that those residues (also Gly294 at Cb-2 [53]) participate in signal transduction by intramolecular contacts rather than by direct intermolecular interactions. Mutations at these positions might induce structural changes and thus modify the arrangement of binding relevant amino acids in close proximity.

Bovine TSH contains positively charged residues at the α-subunit (figure 2A), which are known to increase hormone binding properties compared to hormone variants without these positive charges at corresponding positions such as hFSH and hTSH [70]. There is experimental evidence that these residues interact with the positively charged hormone side chains of bTSH [51], [52] and they are responsible for super-agonistic effects mediated by bTSH compared to hTSH (figure 2A). These complementary charge-charge interactions were already suggested in 2009 (review [2]) by combining functional insights with previous structural implications and have now been substantiated. Indeed, our current (FSHR crystal structure based) TSHR model suggests complementary interactions between these four side chains and the negatively charged side-chains of Asp382 and Glu303 at the N- and C- terminal hinge region.

However, one difference between the β-subunits of bTSH and hTSH cannot be directly explained by the current monomeric TSHR/TSH model. The positively charged Arg89 of the bTSH β-subunit is absent in hTSH and substitution of this residue into hTSH greatly enhances the binding properties of hTSH [70], [71]. The structural model (figure 3) reveals that this residue at the β-L3 loop is located opposite to the second binding region and is not involved in any intra- or intermolecular interaction at this monomeric model. Therefore, the functional effect of this substitution is not explained by a one receptor - one hormone molecule constellation and might point to a functional impact in an oligomeric receptor scenario. This would be in accordance with recent findings at the TSHR, where the receptor-hormone stoichiometry was found not to be symmetric in an oligomeric constellation [47], [72]. Furthermore, it might be speculated that this positively charged residue is a determinant for extracellular trans-activation [59]. This means that not the targeted receptor but a second TSH receptor molecule in close proximity becomes activated by this hormone contact [59].

### An extended and refined scenario of TSHR activation

**Figure 5:**
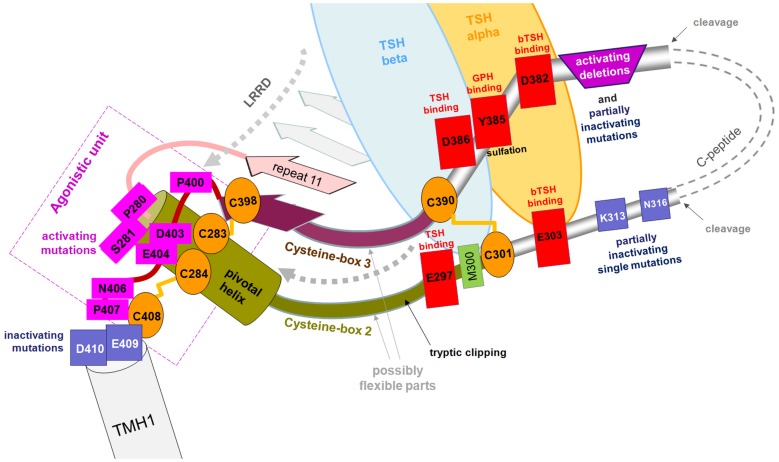
Scheme of initial TSHR activation. New functional-structural TSHR features at the extracellular site are described for the hinge region and the LRRD ain a schematic overview. Repeat 11 with the pivotal helix (green) is part of the LRRD that ends at Asn288 and therefore includes Cys283 and Cys284 of cysteine-box 2 (C-b2). These cysteines are linked by disulfide bridges to Cys398 and Cys408 of cysteine-box 3, respectively. Constitutively activating mutations at Pro280 and Ser281 (magenta) influence the conformation of this helix and underline the importance of this region for receptor function. Further positions of CAMs are located at the C-terminal part of C-b3, in close spatial proximity to Cb-2. Mutations at both fragments probably cause conformational changes at this region which induce receptor activation. Thus, these wild type amino acids constitute an activation-sensitive *intramolecular agonistic unit* (magenta, boxed). This unit is adjacent to TMH1 and is probably located between the ECLs as an interface between the extracellular and transmembrane regions. The transition between the extracellular region and TMH1 is made up of negatively charged amino acids, in which side chain substitutions lead to impaired signaling capacity. These are key to interactions with the serpentine domain spanning the membrane. Several residues identified as sensitive to hormone binding are enclosed in a red box (Glu297, Glu303, Asp382 and Asp386) and represent a spatial cluster that is linked by a disulfide bridge between Cys301 and Cys390. Asp382 (and Glu303) are important for super-agonistic effects of bTSH but are not important for hTSH. Sulfation at Tyr385 is obligatory for hormone binding and is accompanied by negatively charged amino acids close to Tyr385 (Asp386, Asp382). Two cleavage sites define the so-called cleavable peptide (C-peptide, ∼50 amino acids). Deletions at the transition between the C-peptide and the Cb-3 are reported to activate the TSHR (and also the FSHR) constitutively (magenta trapezoid). In contrast, single point mutations lead to partial inactivation of TSHR signaling at this region. The dashed arrows indicate the potential signal processing path for activation upon ligand binding from the hinge region and/or potentially via the LRRD. In either case, the extracellular modifications converge at the pivotal helix that links the *intramolecular agonistic unit* together (dashed box).

We conclude that the new structural information is in good accordance with the experimental data for the TSHR - at least for the monomeric complex. The newly derived TSHR model is also applicable to further aspects of signaling such as antibody binding and action. We summarize our insights for the TSHR as follows:

The bound hormone is fixed at the TSHR LRRD (extended through Asn288) and employs a lever-like mechanism to displace the hinge region by interacting specifically with amino acids at the N- and C-terminal hinge region in spatial proximity to the sulfated Tyr385 (figures 3 and 5). In the case of bTSH, but not hTSH, additional charge-charge interactions are of importance for binding at the second receptor binding site.

Upon hormone binding, this spatial displacement triggers conformational changes at the LRRD and the N- and C-terminus of the hinge region including the pivotal helix of the eleventh repeat and twelfth β-strand at Cb-3. A second signal processing path via rigid body movement of the LRRD cannot be excluded. In either case, the extracellular modifications converge at the pivotal Cb-2 helix. Conformational changes at this helix and structural regions in direct vicinity activate the *intramolecular agonistic unit* close to TMH1. In this context, it is striking that with the exception of the disulfide-bridge Cys229–Cys338 (figure 1) no side chain or backbone interaction can be observed in the FSHR crystal structure that stabilizes the hairpin-like fold of the hinge region between the remaining residues of Cb-2 and Cb-3. One can speculate that this indicates conformational movability of this region in support of the suggested scenario. In accordance with this, it is exactly this region between positively charged residues K290–K293 at the N-terminal hinge region Cb-2 in TSHR that is accessible for tryptic clipping [40] (figure 3). Tryptic clipping also partially activates the TSHR [73]. The hypothesis that displacement of the hinge region is relevant to signaling is further supported for the TSHR by constitutive activation caused by deletions of extracellular regions (e.g. the fragment between positions 371–384 (figure 5) [30]); the corresponding region is not resolved in the FSHR structure. Such constitutive activation has also been reported for the FSHR [26]. This hinge region fragment either indirectly masks or might even be located in direct proximity to the *agonistic unit* (figure 3 and 5) and thus participates in restraining the basal conformation of the receptor.

Moreover, so far it is not understood how the TSHR specific insertion of around 50 amino acids (C-peptide) in the middle of the hinge region [63]–[68] influences the function of the described hinge region segments that are relevant for signaling. One could speculate that its flexibility and functionality might be impeded in a non-cleaved stage by the intact C-peptide and is increased in the cleaved stage. In this regard, it was postulated in a recent study that the occurrence of TSHR cleavage might be dependent on cell–cell contacts since an almost complete cleavage was observed in confluent cells (like in thyroid tissue) while in sparse cells (cultured thyrocytes and non-thyroid cells) most of the TSHR was in an uncleaved form [74]. Furthermore, activation of Gq was found to be reduced in non-cleaved TSHR (cultured cells) [74]. This finding would reveal that presence or absence of cleavage is of potential relevance for simultaneous induction of both Gs and Gq-mediated signaling pathways. Interestingly, the capacity for dual signaling of TSHR was also found to be dependent on the receptor-hormone stoichiometry in an oligomeric constellation [72]. Two TSH molecules bound at a TSHR homodimer are necessary to activate both effectors Gs (cAMP) and Gq (IP) while activation of a TSHR dimer by just one TSH molecule only induces Gs signaling. However, concrete dependencies between the phenomena of cleavage, oligomerization, hormone binding and dual signaling capacity associated with activation mechanisms at the hinge region are not yet provided by direct experimental data, but shall be an aspect of future studies.

Anyhow, the activation-sensitive key participants in the *agonistic unit* (as so far investigated and recognized) are Pro280, Ser281 at Cb-2, and Pro400, Asp403, Glu404, Asn406, Pro407 at Cb-3 and are located in close spatial proximity to each other. They are linked by disulfide bridges (figure 3 and 5). This unit is adjacent to TMH1 and therefore, is also likely to be embedded between the ECLs as an interface between the extracellular and transmembrane region. Receptor activation is, therefore, probably accompanied by modification of the adjustment between the unit-determinants (Cb-2/Cb-3) and the ECLs or their transitions to the helices which is substantiated by known CAMs at ECLs of TSHR (reviewed in [2]). The binding area of activating auto-antibodies also covers the C-terminal LRRD and does not necessarily interact with the sulfated tyrosine at the hinge region. Due to their greater bulk, the antibodies might convey their signal directly to the *intramolecular agonistic unit* close to TMH1 or/and by spatial displacement of further hinge parts that finally affects the above mentioned conformation and precise adjustment of the *agonistic unit* relative to the SD.

Finally, detailed investigation of the cooperative interplay between the SD and the hinge region [46], [47], [55], [59], [75], negative cooperative effects on binding [59], [76]–[78] and aspects of TSHR organization and mechanisms such as trans-activation [59], [79] will progressively benefit from these new insights.

## Materials and Methods

### Molecular homology modeling and bioinformatics

For the design of a hTSHR C-terminal hinge region fragment model (hTSHR positions Ser383-Ile411) the corresponding region in the recently published hFSHR crystal structure (PDB entry code: 4AY0 [54]) was used as a structural template (FSHR positions Thr331-Ile359). In addition, a template is not provided for Asp382, which is located adjacent to Ser383 in the TSHR (figure 2B). It has been observed that mutations at this Asp382 [51], [52] in bTSH (and super-agonistic TSH analog TR1401, Trophogen Inc., Rockville, MD) impaired the hormone binding and signaling capacities of hTSHR in contrast to stimulation with hTSH. This observation supports interactions between Asp382 and particular positively charged amino acid side-chains in the bTSH α-subunit that are absent in hTSH and hFSH (figure 2B). These residues at the bTSH α-subunit are Lys39, Lys41, Lys44 and Lys48. Asp382 was therefore added to the current initial TSHR homology model of bTSH/hTSHR, and orientated in close proximity to the accessible hormone αLys39 and αLys41 as a functional constraint.

At the C-terminal GPHR hinge region specific small differences do exist. Most importantly, the sulfated FSHR Tyr335 already known as mandatory for hormone binding and signaling - interacts tightly with amino acids of the hormone subunits between the β-subunit loop 2 and the α-loop 1 [54]. The tyrosines of TSHR and LHR at the corresponding positions are not sulfated (figure 3), but (as shown experimentally) the tyrosines two positions upstream are sulfated (in TSHR Tyr385, LHR Tyr331) and are obligatory for full signaling functions [49], [62]. Therefore, assuming that this sulfated tyrosine in TSHR also interacts with the same hormone binding site as that observed for the FSHR/FSH complex, there may be slight structural differences around Tyr385. In consequence, in the TSHR model Tyr385 substitutes for the sulfated FSHR Tyr335.

The incomplete LRRD of hTSHR with 9 repeats was extracted from the LRRD/antibody complex [22] (PDB entry code: 3G04) and extended by adding repeat amino acids from the recent full FSHR LRRD crystal structure with 11 repeats [54].

Consecutively to Cb-2, the FSHR LRRD crystal structure included the N-terminal fragment of the hinge region (FSHR positions Trp291-Ser295, TSHR positions Gln289-Ser304). This portion was also added to the TSHR LRRD model and side-chains of TSHR were substituted. Disulfide bridges between cysteines of Cb-2 and Cb-3 were built as suggested by the FSHR crystal structure (figures 1 and 2).

The hormones bTSH, hTSH and thyrostimulin were modelled based on FSH (sequence alignment provided in figure 2A), whereby the mode of orientation between hormones and LRRD were maintained in the models of the complex as suggested by the FSHR/FSH complex. In consequence, the activated LRRD-hinge region-hormone complexes postulated by the FSHR LRRD/FSH structure were designed and used for comparison.

Structural modifications and homology modeling procedures were performed with Sybyl X2.0 (Tripos Inc., St. Louis, Missouri, 63144, USA). Side-chains of the homology models were subjected to conjugate gradient minimizations (until converging at a termination gradient of 0.05 kcal/(mol*Å)). The AMBER F99 force field was used. Finally, the models were minimized without constraints.

Structure images were produced using PyMOL software (DeLano WL, version 1.03, San Carlos, CA, USA).
